# Students’ stress prediction and explainable analysis based on improved decision trees

**DOI:** 10.3389/fpsyg.2025.1684529

**Published:** 2026-01-02

**Authors:** Cheng Liu, Shuang Yu

**Affiliations:** Department of Digital Business, Jiangsu Vocational Institute of Commerce, Nanjing, Jiangsu, China

**Keywords:** student stress prediction, decision tree algorithm, harris hawks optimization, SHAP model, machine learning

## Abstract

**Introduction:**

Nowadays students are burdened with pressures from various aspects such as academics, social life, and career planning. It is of great significance to accurately predict their stress levels and analyze the key influencing factors.

**Methods:**

A stress prediction model for students was constructed based on an enhanced decision tree (DT) algorithm. First, nine machine learning algorithms, including logistic regression (LR) and DT, were compared to screen out the optimal base model. Then, the harris hawks optimization (HHO) algorithm was introduced to optimize the DT model and improve its prediction performance. Finally, the Shapley Additive Explanations (SHAP) model was applied to interpret the prediction results and analyze the contribution of various features to stress levels.

**Results:**

The DT algorithm showed outstanding performance among the nine compared models, achieving a prediction accuracy of 0.909. After optimization by the HHO algorithm, the HHO-DT model further improved the accuracy to 0.927 and had the fewest misclassified samples. SHAP analysis revealed that blood pressure, social support, and depression were the key features affecting students’ stress level prediction.

**Discussion:**

The research results provide a scientific and effective basis for intervention measures taken by mental health educators, parents, and students themselves, which is helpful to relieve students’ stress and promote their physical and mental health.

## 1 Introduction

In today’s society, adolescents and young adult students (AYAS) are burdened by pressures from various aspects, including academics, social life, and career planning. Academically, with the increasingly fierce social competition and the ever-changing educational environment, AYAS face unprecedented psychological and life pressures (Harahap et al., 2022). In addition to academic stress, they are also troubled by issues related to social and interpersonal relationships. These pressures may arise from peer relationships, teacher-student interactions, and perceptions of self-identity, among other factors (Guo R., 2024). Research has shown that a lack of social support can further exacerbate students’ psychological burdens (Jia and Chu, 2024). Moreover, when navigating interpersonal relationships, AYAS encounter numerous challenges, including emotional problems and social expectations (Chen, 2013). Prolonged exposure to such pressures makes AYAS highly susceptible to mental health issues, with symptoms of depression being particularly prevalent. A study on AYAS found that daily hassles are more likely to trigger depressive emotions than major life events (Shin, 2024).

Recent studies have shown that combining metaheuristic optimization algorithms with traditional machine learning models can significantly improve classification accuracy and reduce computational costs. For example, the combination of greylag goose optimization (GGO) and a multi-layer perceptron achieved an accuracy of 98.4% in lung cancer classification (Elkenawy et al., 2024), while the gradient boosting model optimized by binary particle swarm optimization (BPSO) reached a precision of 95.5% in the prediction of COVID-19 transmission (Alkhammash et al., 2023). The combination of deep learning models such as convolutional neural networks (CNN) and long short-term memory networks (LSTM) has also made breakthroughs in agricultural disease detection. For instance, the CNN-LSTM model achieved a remarkably high accuracy of 97.1% in the identification of potato late blight, demonstrating its advantages in time-series data and image analysis (Alzakari et al., 2024). However, existing methods still face challenges such as high computational complexity, the risk of overfitting, and feature redundancy. There is an urgent need for more efficient optimization strategies. The crucial role of optimization algorithms in feature selection and model parameter tuning provides the inspiration for this study.

In this context, accurately predicting the stress levels of AYAS and analyzing the key influencing factors have become pressing issues that warrant urgent attention. This research aims to construct a predictive model for AYAS stress using an enhanced DT algorithm. The objective is not only to achieve precise identification of AYAS’ stress states but also to provide an interpretable analysis that elucidates the mechanisms through which various factors influence stress levels. This research offers a scientific and effective foundation for interventions by mental health educators, parents, and the students themselves, thereby contributing to the alleviation of stress and the promotion of physical and mental health among AYAS. The innovations of this paper are as follows:

- Through comparing nine machine learning algorithms, the DT model demonstrated the optimal performance in predicting student stress with an accuracy of 0.8955 and an F1 score of 0.8952. And, paired *t*-test validation confirmed its superiority over models such as Random Forest (RF) and XGBoost (XGB).
- Introducing the HHO-DT model with accuracy of 0.927, while achieving a runtime of only 26.9 s, outperforming grid search of 117.2 s.
- Feature impact quantification via SHAP model revealed blood pressure, social support, and depression as core factors in stress prediction, enhancing model transparency and practical value while providing a scientific basis for intervention measures.

## 2 Literature review

### 2.1 Research of student stress

In today’s learning environment, students encounter various forms of stress, with academic stress being one of the most significant influencing factors. The increasing difficulty of courses, the growing volume of homework, the frequent administration of exams, and the escalating competition for further education all impose a considerable burden on students (Al-Rouq et al., 2022). A study (Chust-Hernández et al., 2022) has highlighted that for nursing students, school-related factors significantly impact their academic stress. Tasks such as classroom presentations and insufficient time to complete assignments are particularly important sources of stress.

Moreover, family-related factors should not be overlooked. Parents’ excessive expectations, the quality of family relationships, and the family’s economic situation can all influence students’ stress levels to varying degrees (Nguyen et al., 2018). For instance, literature (Hossain et al., 2023) clearly indicates that economic factors, such as tuition arrears and borrowing, are positively correlated with the economic stress experienced by college students. In the social realm, the challenges students face in managing relationships with classmates and teachers, as well as the difficulties in integrating into social groups, can also contribute to their stress levels (Bartlett et al., 2016). Additionally, individual factors play a crucial role in how stress is perceived and managed. A study (Maykrantz and Houghton, 2020) has demonstrated that elements such as personality, psychological resilience, and self-perception can influence students’ perceptions of and responses to stress. This research further indicates that self-leadership and coping skills can help regulate students’ stress levels, underscoring the significant impact of individual psychological factors on stress management.

Long-term exposure to stress can negatively impact students’ physical and mental health. A study of German students (Kiupel et al., 2023) found an association between stress and skin itching, suggesting that stress may adversely affect physical well-being. From an academic perspective, moderate stress can be transformed into motivation for learning; however, excessive stress can hinder the learning process. Taking Thai medical students as an example (Saipanish, 2003), the study highlights that the stress they experience is closely linked to academic challenges, implying the detrimental effects of excessive stress on academic performance. Additionally, other literature (Ragab et al., 2021) underscores the adverse effects of academic stress on medical students.

To effectively alleviate students’ stress, intervention measures must be implemented at multiple levels. At the school level, optimizing the curriculum, actively developing mental health education courses, and fostering a positive campus atmosphere can significantly help reduce students’ stress (Nguyen-Thi, 2024). One study (Antoniadou et al., 2024) suggests that schools should enhance their support systems and refine academic procedures to better address students’ stress-related issues. The family environment also plays a crucial role. Parents who adjust their expectations, adopt democratic educational methods, and provide ample emotional support can positively influence their children’s ability to cope with stress. Although relevant literature does not extensively detail family intervention measures, the significant impact of family factors on students’ stress indirectly underscores the importance of such measures. For students themselves, acquiring effective coping skills and cultivating a diverse range of hobbies can enhance psychological resilience, thereby mitigating stress. For example, a study (Mahoney and Bussard, 2024) has shown that intervention measures such as relaxation training can reduce the stress levels of nursing students, which fully reflects the importance of students’ self-regulation in the process of coping with stress.

### 2.2 Research of machine learning in predicting student stress

Research on the application of machine learning to predict student stress is gaining increasing attention. Numerous studies focus on enhancing prediction accuracy through various algorithms and data sources, thereby providing robust support for interventions in students’ mental health.

Regarding the construction of prediction models and the comparison of algorithms, many studies have achieved notable results. In El Morr et al. (2024), eight machine learning models were developed to assess the depression, anxiety, and stress symptoms of Lebanese university students during the COVID-19 pandemic. The study found that the random forest (RF), naive bayes (NB), and adaboost models excelled in predicting these symptoms. In the study referenced as Ghosh et al. (2024), researchers created multiple algorithms to predict changes in student stress following yoga practice, with the RF algorithm demonstrating the lowest root mean square percent error (RMSPE) and a relatively high r-squared value. Schaab et al. (2024) conducted a systematic review of machine learning models for detecting depression, anxiety, and stress in undergraduate students, revealing that the accuracy of most models exceeded 70%. However, the review also noted issues such as over-reliance on internal validation and low-quality evidence. These studies indicate that different algorithms possess distinct advantages and disadvantages in various scenarios, offering a valuable reference for subsequent research in selecting appropriate algorithms. Notably, Arya et al. (2024) uses the same dataset as the present study, but relies on the GridSearchCV method for hyperparameter optimization. Its performance is constrained by the design of search space and step size, resulting in low efficiency. Furthermore, its analysis is limited to correlation coefficients and confusion matrices, lacking quantitative interpretation of the contribution of features to predictions. In contrast, the present study introduces a metaheuristic optimization framework and adopts the HHO algorithm to optimize the hyperparameters of the DT model, enabling efficient search of the parameter space. In addition, the present study integrates the SHAP model, and quantifies the degree of influence of each feature on the prediction results by calculating the average SHAP value of each feature, which provides an operable scientific basis for campus mental health educators and parents to formulate targeted intervention measures.

Data sources and feature selection are crucial for predicting stress. Shvetcov et al. (2024) utilized passive sensing data from smartphones, including GPS and step-counting data, to predict stress levels among university students and established an effective methodological process. Liao et al. (2024) selected factors such as sleep and exercise for feature extraction based on intelligent sensing data and psychological stress scales, constructing a psychological stress assessment model for university students. Zhang et al. (2024) integrated multi-source data (psychological, lifestyle, and exercise data) to predict the risk of sleep disorders in university students, identifying key features such as stress scores and the severity of depression. This demonstrates that the integration of multi-source data can provide more comprehensive information for stress prediction and enhance prediction accuracy.

Research on predicting stress in specific student groups has also progressed. In the study referenced as Rahman and Kohli (2024), focusing on international students, a model was developed using online surveys and existing datasets to predict their depression, achieving an accuracy rate of 80% with the RF model. Kong et al. (2025) combined multiple machine learning methods to predict the severity of mental health issues among Chinese freshmen in universities, discovering that factors related to interpersonal relationships had the strongest predictive power. Calderon et al. (2024) employed machine learning and Bayesian networks to analyze a sample of American university students, revealing the connection between insomnia and mental disorders, thereby providing a basis for relevant interventions. These studies have yielded targeted strategies for stress management in student groups with diverse backgrounds.

Although machine learning has yielded promising results in predicting student stress, it continues to encounter several challenges. The quality of the data is inconsistent, with issues such as missing values and noise that adversely affect model performance. Furthermore, the generalization ability of these models requires enhancement, and their stability across diverse student groups and scenarios remains inadequate. Additionally, ethical concerns, including data privacy protection and algorithmic fairness, must also be addressed.

### 2.3 Research of intelligent optimization

The research on the application of intelligent optimization algorithms in numerous fields is continuously advancing in depth. Among them, the HHO, BES, GWO, SSA, and particle swarm optimization (PSO) have been developing vigorously. These algorithms, with their unique search mechanisms, provide effective solutions to complex optimization problems.

Numerous scholars have conducted extensive research and innovative practices on these algorithms, yielding fruitful results. In Tang et al. (2024), researchers proposed an improved harris hawks optimization algorithm (IHHO) integrated with a back propagation (BP) neural network, applying it to high-precision landslide displacement prediction. Practical experiments demonstrate that the IHHO-BP model possesses significant advantages in addressing complex nonlinear problems. This model greatly enhances prediction accuracy and provides crucial technical support for landslide disaster warning. Zaky et al. (2021) applied the BES algorithm to the modeling and simulation of perovskite solar cells (PSCs). The study revealed that the BES algorithm exhibits high accuracy and efficiency in determining the device parameters of PSCs, particularly when addressing complex nonlinear issues, thereby opening new avenues for research and development in perovskite solar cells.

In the field of healthcare, Tarek et al. (2025) applied the snake optimization algorithm (SO) to the detection of cardiovascular diseases, and achieved a high precision of 99.9%, demonstrating the role of optimization algorithms in reducing dimensionality and enhancing the generalization ability of models. The improved Al-Biruni Earth Radius (MBER) algorithm adjusted the exploration and exploitation strategies dynamically. It achieved an accuracy of 96.12% in the classification of eye movements from electroencephalogram (EEG) signals (Elshewey et al., 2024), providing new ideas for biomedical signal processing.

In the field of mechanical engineering optimization, the GWO model exhibits exceptional performance. Yildiz et al. (2020) applied the GWO to various mechanical engineering optimization problems and compared its efficacy against multiple meta-heuristic algorithms. The results substantiate the GWO’s superior performance, highlighting its significant application value in the optimization design of mechanical engineering. The SSA also demonstrates robust capabilities. Xue and Shen (2020) introduced the SSA and conducted comprehensive tests of its performance using 19 benchmark functions. The results indicate that, in most instances, the SSA outperforms classic algorithms such as the GWO and PSO. To further enhance the SSA’s performance, Guo W. (2024) introduced a learning mechanism, resulting in the learning-based SSA (LSSA), which effectively addresses the issue of the traditional SSA’s tendency to converge to local optima. Lastly, Ye et al. (2022) proposed a feature selection method based on PSO. By integrating a differential evolution strategy, this method greatly improves the global search capability of the algorithm, allowing the PSO to more accurately identify key features in the feature selection task, thus enhancing the performance of related models.

Overall, intelligent optimization algorithms hold significant potential for addressing complex optimization problems. Algorithms such as HHO, BES, GWO, SSA, and PSO each possess unique advantages, making them suitable for various application scenarios. Ongoing in-depth research and enhancements of these algorithms contribute to a better understanding of their performance advantages and facilitate the expansion of their application domains.

## 3 Materials and methods

### 3.1 Data description

The dataset utilized in this study was sourced from Kaggle and targets a student population with extensive coverage. And, the dataset was primarily collected from high school and college students in Dharan, Nepal. Participants were aged between 15 and 24 years, representing the adolescent and young adult population typically studied in stress research. The data collection period spanned from June to October 2022, using stratified sampling to ensure representation across different grades and academic disciplines. Comprising 1,100 samples, this dataset includes 20 variables related to student stress, spanning multiple dimensions: psychological, physiological, social, environmental, and academic. As presented in Table 1, the mean (MEAN) and standard deviation (STD) were calculated for each variable, accompanied by a clear interpretation of each field’s significance. The mean value of student’s stress levels is 1.51, which falls within the upper-middle portion of the range [0, 2], indicating that students generally experience a relatively high level of stress. The mean value of anxiety levels is 13.95, also situated in the upper-middle range [0, 21], suggesting that students, overall, exhibit a certain degree of anxiety. Additionally, the mean value for mental health history is 0.69, indicating that approximately 69% of students have a history of mental health issues, which represents a notably high proportion. By analyzing this dataset, we can verify the impact of factors such as Psychological, Physiological, Environmental, Academic, and Social on students’ stress, thereby addressing the stress issue in a more targeted manner.

**TABLE 1:** Statistics and explanations of stress indicators.

Field name	Mean	STD	Description
anxiety_level	13.95	4.65	0–21, higher values indicate higher anxiety levels
self_esteem	13.94	7.62	0–30, higher values indicate stronger self-esteem
mental_health_history	0.69	0.46	1 = history of mental health issues, 0 = no
Depression	15.91	6.35	0–27, higher values, more severe depression
Headache	3.12	1.18	0–5, higher values, more frequent headaches
blood_pressure	2.17	0.99	1–3, higher values, more serious blood problems
sleep_quality	1.91	1.12	0–5, higher values, better sleep quality
breathing_problem	3.3	1.26	0–5, higher values, severe breathing issues
noise_level	3.16	1.17	0–5, higher values, louder environmental noise
living_conditions,	2.11	0.96	0–5, higher values, better living conditions
Safety	2.04	0.93	0–5, higher values, greater safety perception
basic_needs	2.07	0.98	0–5, higher values, better satisfaction of basic needs
academic_performance	2.07	0.95	0–5, higher values, better academic performance
study_load	3.12	1.19	0–5, higher values, heavier study loads
teacher_student relationship	1.99	0.85	0–5, higher values, better teacher-student relationships
future_career concerns	3.32	1.3	0–5, higher values, greater concern about future careers
social_support	1.54	0.93	0–3, higher values, stronger social support
peer_pressure	3.28	1.32	0–5, higher values, more intense peer pressure
extracurricular_activities	3.31	1.3	0–5, higher values, more frequent participation in extracurricular activities
bullying	3.32	1.29	0–5, higher values, more severe bullying experiences
stress_level	1.51	0.5	0–2, higher values, higher stress levels (target variable)

The core psychological indicators in the dataset were measured using internationally recognized standardized scales with clear operational definitions and scoring ranges: *anxiety_level* was assessed by the Generalized Anxiety Disorder-7 Scale (GAD-7, scoring range 0–21); *self_esteem* was measured by the Rosenberg Self-Esteem Scale (scoring range 0–30); and *depression* was evaluated by the Patient Health Questionnaire-9 (PHQ-9, scoring range 0–27). The Cronbach’s α coefficient for the mental health dimension is 0.649, which falls within the acceptable range. For other non-psychological scale indicators detailed in Table 1, their scoring follows a unified logical framework, scores of 0–1 represent “low level,” 2–3 represent “moderate level, and 4–5 represent “high level,” with physiological indicators like *blood_pressure* graded with reference to conventional clinical assessment standards, ensuring the indicator values have practical significance and discriminability to characterize students’ physical status and living environment.

**FIGURE 1:**
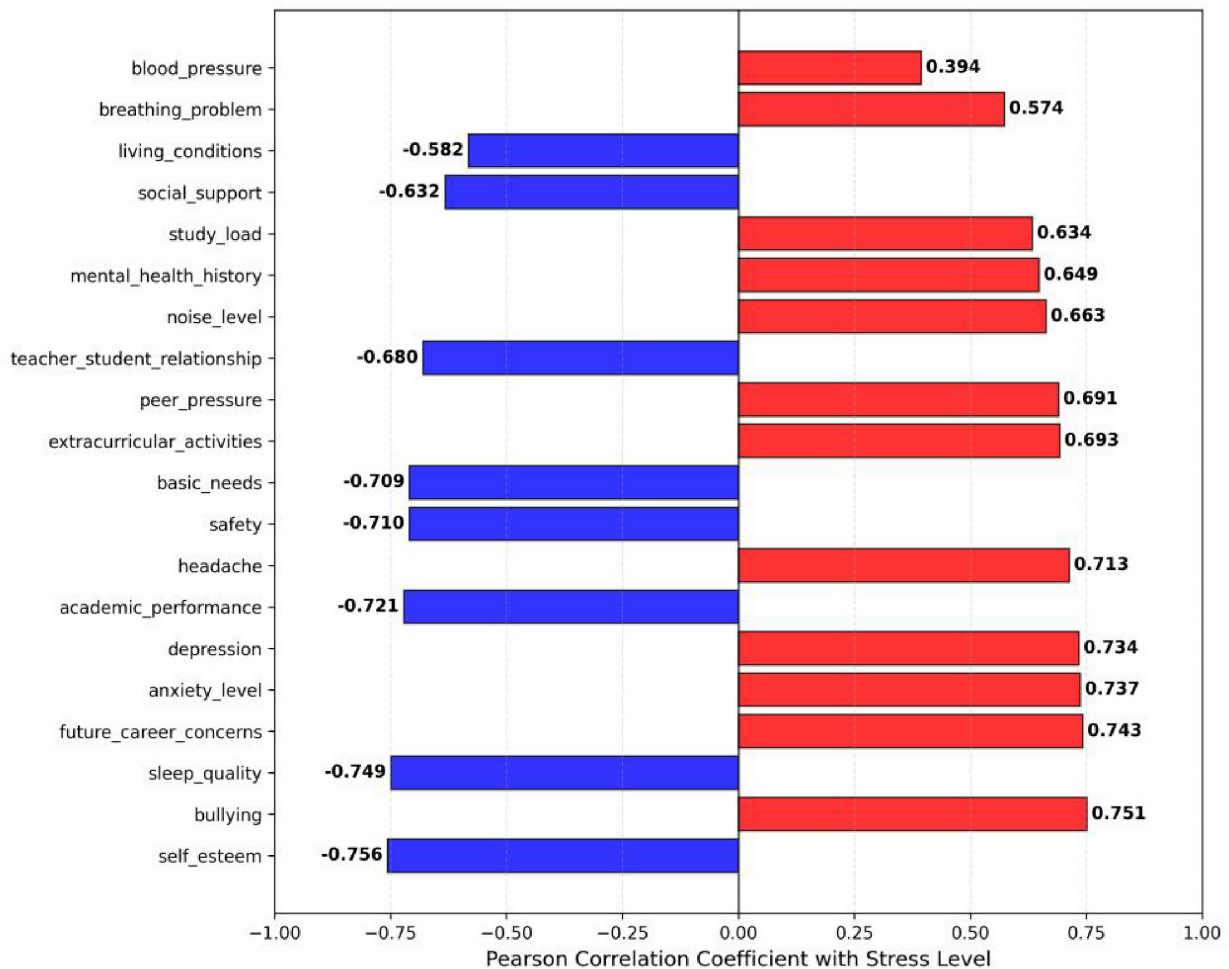
Correlation between stress level and other variables.

Preliminary statistical checks confirmed that all samples had complete data across the 21 dimensions, with all values falling within reasonable ranges. Therefore, the dataset can be directly used for modeling analysis without additional preprocessing. To investigate the factors influencing stress level, Pearson correlation coefficients between stress level and the other 20 variables were calculated. As shown in Figure 1, except for blood_pressure, the absolute values of the correlation coefficients for all other variables exceed 0.5, indicating moderate to strong associations with stress level and justifying their inclusion in further analysis.

### 3.2 Decision tree algorithm

The DT model is a commonly used machine learning algorithm, which is widely applied to classification and regression tasks (Chen et al., 2011). It simulates the human decision making process. By analyzing and judging the features of data, it gradually divides the data set into smaller subsets until each subset belongs to the same class as much as possible.

Step 1, in the feature selection stage, an optimal feature is selected from the current data set for partitioning the data set. Commonly used indicators include information gain. The larger the information gain, the better the feature can reduce the uncertainty of the data set when used for partitioning, that is, the greater the contribution of this feature to classification.

Suppose the data set *D* contains *n* classes, and the number of samples in the *i*-th class is |*C*_*i*_| . Then the entropy of the data set is defined as in Equation 1:


$$H\,\left(D\right)=-\sum_{i=1}^{n}\frac{\left|C_{i}\right|}{\left|D\right|}\,\log_{2}\,\left(\frac{\left|C_{i}\right|}{\left|D\right|}\right)$$ (1)

In which, |*D*| represents the total number of samples. When the data set *D* is partitioned using the feature A, the information gain is defined as in Equation 2:


$$G\,\left(D,A\right)=H\,\left(D\right)-\sum_{j=1}^{v}\frac{\left|D_{j}\right|}{\left|D\right|}\,H\,\left(D_{j}\right)$$ (2)

Among them, |*D*_*j*_| represents the total number of samples in the *D_j_* dataset, and *H*(*D*_*j*_) represents the entropy of the *D_j_* dataset . The larger the information gain, the greater the contribution of feature A to classification.

Step 2, based on step 1, partition the data set. Divide the current data set into several sub-data sets, with each sub-data set corresponding to one value of the feature.

Step 3, recursively construct sub-trees, for each sub-data set, repeat the above processes of step 1 and step 2, until the samples in the sub-data set all belong to the same class or there are no more features available for selection.

### 3.3 HHO algorithm

The HHO model is a novel metaheuristic optimization algorithm inspired by the cooperative hunting behavior of harris hawks in nature (Heidari et al., 2019). It has shown remarkable performance in solving a wide variety of complex optimization problems due to its efficient exploration and exploitation capabilities. During the exploration phase, the hawks search for potential prey over a wide area, mimicking the global search behavior in optimization. In the exploitation phase, once the prey is spotted, they use different tactics to capture it, similar to the local search refinement in optimization algorithms.

Let $X_{i}^{t}$ denote the position vector of the *i*-th hawk in the *t*-th iteration. In the exploration phase, the position update rule is designed to encourage the hawks to explore different regions of the search space. The equation is given in Equation 3:


$$X_{i}^{t}=X_{r\,a\,n\,d}^{t}-r_{1}*\left|C*X_{r\,a\,n\,d}^{t}-X_{i}^{t}\right|$$ (3)

In which, $X_{\text{rand}}^{t}$ is a randomly selected hawk’s position from the current population at iteration, *r*_1_ is a random number in the range [0, 1], and *C* is a constant coefficient in the range between 0 and 2.

During the exploitation phase, when the hawks have identified a promising region (prey location), the position update is more focused on local search. There are multiple strategies depending on the fitness of the hawks and the stage of the search. One of the commonly used equations is given in Equation 4:


$$X_{i}^{t}=X_{b\,e\,s\,t}^{t}-2*E_{0}\,\left(1-\frac{t}{T_{m\,a\,x}}\right)*\left|J*X_{b\,e\,s\,t}^{t}-X_{i}^{t}\right|$$ (4)

Where $X_{\text{best}}^{t}$ is the position of the best performing hawk at iteration *t*, *E*_0_ is the initial escape energy, usually set to a value between 1 and 2, *t* is the current iteration number, and *T*_*max*_ is the maximum number of iterations, and *J* is a random number between −1 and 1.

This study introduced the HHO algorithm, aiming to meticulously optimize the hyperparameters of the DT algorithm, thus effectively enhancing the accuracy and reliability of the model and laying a solid foundation for subsequent precise pressure classification.

**FIGURE 2:**
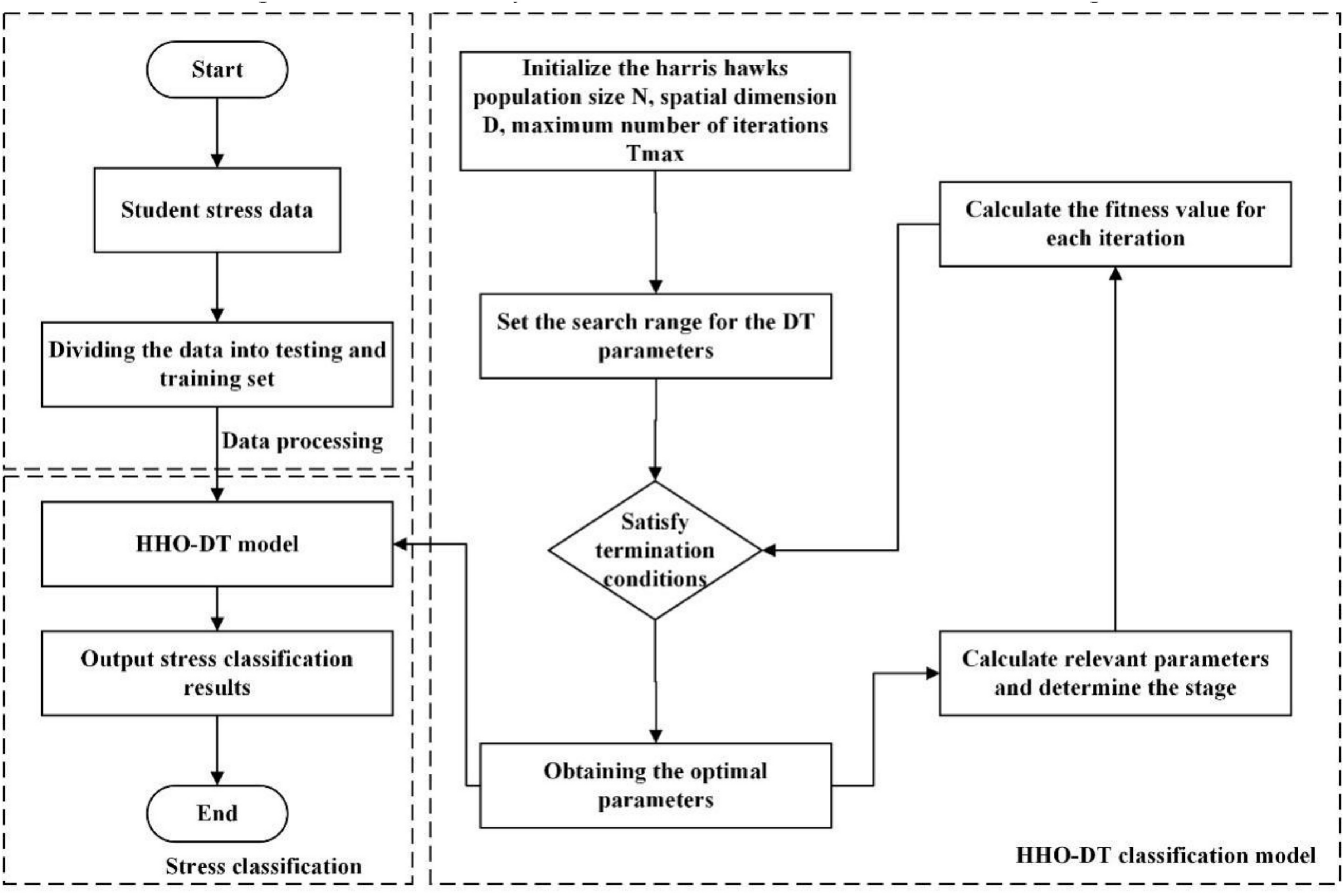
Work flow of HHO-DT model.

The dataset was divided into a training set and a testing set at a ratio of 4:1. On the training set, the HHO algorithm was employed to optimize hyperparameters of the DT to determine the optimal parameters of the model. And, the determined optimal model was used to predict the testing set to further validate the generalization ability of the model, the work flow is shown in Figure 2.

### 3.4 Interpretive SHAP model

The SHAP model is a unified approach for interpreting the prediction results of machine learning models (Hancock et al., 2025). It is based on the concept of Shapley values in cooperative game theory and aims to provide a reasonable and unique attribution for the contribution of each feature to the model’ prediction. In this study, we applied the SHAP model to explain the student stress prediction model, in order to deeply understand the impact of each feature on the prediction results.

Specifically, for a dataset X containing n features and a machine learning model f, the Shapley value ϕ_*i*_ of feature *i* is defined as in Equation 5:


$$\phi_{i}=\sum_{S\subseteq N\backslash \left\{i\right\}}\frac{\left|S\right|!\,\left(n-\left|S\right|-1\right)!}{n!}\,\left[f\,\left(S\cup \left\{i\right\}\right)-f\,\left(S\right)\right]$$ (5)

Among them, *N* represents the set of all features, S is an arbitrary subset of *N* that does not contain feature *i*, |*S*| indicates the size of subset *S*, and f(S) represents the prediction result of the model when only considering the features in subset *S*.

In the student stress prediction model, by calculating the SHAP value of each feature, we can quantify the degree of influence of each feature on predicting students’ stress levels. A positive SHAP value indicates that the feature tends to increase the predicted stress level, while a negative SHAP value means that the feature tends to decrease the predicted stress level. The larger the absolute value of the SHAP value, the greater the impact of the feature on the prediction result.

### 3.5 Model evaluation metrics

To comprehensively evaluate the performance of the student stress prediction model, this study has selected a series of representative evaluation indicators, including accuracy, precision, recall, and F1 score. These indicators, considered from various dimensions, provide a thorough assessment of the model’s performance in classifying faults and non-fault samples, thereby establishing a robust basis for accurately measuring the model’s reliability, stability, and generalization.

Accuracy intuitively reflects the overall correctness of the model’s predictions by calculating the proportion of correctly predicted samples in the total number of samples. The formula for accuracy is given in Equation 6:


$$A\,c\,c\,u\,r\,a\,c\,y=\frac{T\,P+T\,N}{T\,P+T\,N+F\,P+F\,N}$$ (6)

In which, TP refers to the number of faulty samples correctly identified by the model. TN represents the number of non-faulty samples accurately determined by the model. FP indicates the number of normal samples misjudged as faulty by the model. FN is the number of faulty samples misclassified as normal by the model.

Precision measures the proportion of true positive samples among the samples predicted as positive. It reflects the accuracy of the model in predicting positive examples, that is, how many of the positive examples predicted by the model are actually positive. Its calculation formula is given in Equation 7:


$$P\,r\,e\,c\,i\,s\,i\,o\,n=\frac{T\,P}{T\,P+F\,P}$$ (7)

Recall measures the proportion of actual positive examples that are correctly predicted as positive. It reflects the ability of the model to find all positive examples, that is, how many true positive examples are found by the model. The calculation formula is given in Equation 8:


$$R\,e\,c\,a\,l\,l=\frac{T\,P}{T\,P+F\,N}$$ (8)

The F1 score is the harmonic mean of precision and recall. It integrates the two metrics of precision and recall, and is used to balance and comprehensively evaluate the performance of a model. The higher the F1 score, the better the overall performance of the model in terms of both precision and recall, as shown in Equation 9.


$$\mathrm{F1}\,\mathrm{score}=\frac{2*P\,r\,e\,c\,i\,s\,i\,o\,n*R\,e\,c\,a\,l\,l}{P\,r\,e\,c\,i\,s\,i\,o\,n+R\,e\,c\,a\,l\,l}$$ (9)

## 4 Experimental verification and analysis

### 4.1 Evaluation of multiple machine learning algorithms

In this multi-class classification task of student stress, in order to comprehensively evaluate the performance of different models, multiple machine learning models such as LR, DT, Random Forest (RF), Support Vector Machine (SVM), K–Neighbors (KNN), XGB, LightGBM (LGBM), Gradient Boosting (GBM), and Extra Tree (ET) were used for comparative analysis. The dataset was divided into a training set and a test set. We employed five-fold stratified cross-validation on the training set for hyperparameter tuning to determine the optimal parameters, and finally validated the model on the test set. The search ranges and optimal parameters for each algorithm are presented in Table 2.

**TABLE 2:** Grid search parameters and optimal values for each model.

Algorithm	Parameters	Search space	Best value
LR	C	[0.1,1,3,5,7,9,10]	1
	Penalty	[‘l1’, ‘l2’]	‘l2’
DT	max_depth	[3,5,7,9,18]	20
	min_samples_split	[2,5,7,9]	6
RF	n_estimators	[100,120,140,160,180, 200]	120
	max_depth	[None,3,5,7,9,11]	9
	min_samples_split	[2,4,6]	4
SVM	C	[0.1, 1, 3,5,7,9,10]	7
	Kernel	[‘linear’, ‘rbf’]	‘linear’
KNN	n_neighbors	[3,5,7,9,11]	3
	Weights	[‘uniform’, ‘distance’]	’uniform’
XGB	max_depth	[3,5,7,9,11]	3
	learning_rate	[0.01, 0.1]	0.01
	n_estimators	[100,120,140,160,180, 200]	180
LGBM	num_leaves	[30,40,50,60]	40
	learning_rate	[0.01, 0.1]	0.1
	n_estimators	[100,120,140,160,180,200]	100
GBM	max_depth	[3,5,7,9,11]	5
	learning_rate	[0.01, 0.1]	0.1
	n_estimators	[100,120,140,160,180, 200]	100
ET	n_estimators	[100,120,140,160,180, 200]	100
	max_depth	[None,3,5,7,9,11]	7
	min_samples_split	[2, 5]	5

Ultimately, the performance comparison results of nine machine learning algorithms are presented in Table 3 The DT model demonstrates the best performance in student stress prediction, achieving an accuracy of 0.8955, precision of 0.8967, recall of 0.8955, and F1 score of 0.8952, which are significantly higher than other models. This indicates that DT effectively captures nonlinear relationships in the data through hierarchical feature partitioning, showcasing stronger feature selection capabilities in stress classification tasks. The SVM model follows closely with an accuracy of 0.8909 and an F1 score of 0.8909, approaching the performance of the DT model and demonstrating balanced classification ability for positive and negative samples. LR model and ET model achieve accuracies of 0.8864 and 0.8818, respectively, placing them at a moderate level. LGBM and GBM model both achieve an accuracy of 0.8773, highlighting certain limitations of traditional gradient boosting methods in this task.

**TABLE 3:** Evaluation metrics of multiple machine learning algorithms.

Algorithms	Accuracy	Precision	Recall	F1 score
LR	0.8864	0.8865	0.8864	0.8864
DT	0.8955	0.8967	0.8955	0.8952
RF	0.8773	0.8797	0.8773	0.8777
SVM	0.8909	0.8912	0.8909	0.8909
KNN	0.8727	0.8747	0.8727	0.8730
XGB	0.8636	0.8652	0.8636	0.8639
LGBM	0.8773	0.8795	0.8773	0.8774
GBM	0.8773	0.8781	0.8773	0.8776
ET	0.8818	0.8829	0.8818	0.8821

Because of the randomness in feature selection within the DT algorithm, its accuracy can be affected. To further examine the comparison between the DT model and other models, we used a paired *t*-test. Through 20 rounds of experiments, we obtained the corresponding accuracy values and carried out a comparative analysis with other models. The experimental results are presented in Table 4. All *p* < 0.05, indicating that the accuracy of DT is better than that of other models. In the context of small datasets, the integrated characteristics and parameter sensitivity of XGB make it difficult for it to fully display its advantages, and the difference is the most significant (*t* = 24.07, *p* = 3.85 × 10^−15^).

**TABLE 4:** *T*-test results of DT with other models.

Comparison pair	*t*-statistic	*p*-value
DT-LR	12.92	1.53E–10
DT-RF	10.26	1.43E–11
DT-SVM	5.19	4.88E–05
DT-KNN	16.63	2.26E–12
DT-XGB	24.07	3.85E–15
DT-LGBM	16.61	2.31E–12
DT-GBM	16.61	2.31E–12
DT-ET	8.68	1.46E–09

Table 5 shows the accuracy of various machine learning algorithms for different student stress categories (Stress-0, Stress-1, Stress-2). In the Stress-0 category, RF performed optimally with an accuracy of 0.9211, DT followed closely with an accuracy of 0.9079, indicating strong ability to identify students in the low-stress group. For the Stress-1 category, the DT model excelled with an accuracy of 0.9178, outperforming other models. Both the RF and LR models achieved an accuracy of 0.8493, ranking lowest and demonstrating limited identification capabilities for the moderate-stress group. In the Stress-2 category, SVM showed the best performance, while the DT and ET models both achieved an accuracy of 0.8451, indicating average identification capabilities.

**TABLE 5:** Accuracy of different models for student stress categories.

Algorithms	Stress categories		
	Stress–0	Stress–1	Stress–2
LR	0.8947	0.8493	0.9014
DT	0.9079	0.9178	0.8451
RF	0.9211	0.8493	0.8873
SVM	0.8816	0.8767	0.9296
KNN	0.8553	0.8767	0.8873
XGB	0.8684	0.8904	0.8310
LGBM	0.8421	0.8767	0.9155
GBM	0.8684	0.8630	0.9014
ET	0.8684	0.8904	0.8451

### 4.2 Evaluation of multiple intelligent optimization algorithms

In this study, when optimizing the DT using multiple intelligent optimization algorithms (BES, SSA, GWO, WOA, HHO), the key parameters to be optimized are the values of *max_depth, min_samples_leaf, max_features, min_weight_fraction_leaf, and ccp_alpha*.

In DT, the *max_depth* parameter represents the maximum depth of the DT, that is, the number of nodes on the longest path from the root node to the leaf node. This parameter is used to control the complexity of the DT, and the search range is from 3 to 20. And the *min_samples_leaf* parameter refers to the minimum number of samples required in a leaf node, and the search range is from 0.01 to 0.2. The *max_features* parameter represents the percentage of the maximum number of features considered during each node split, and the search range is from 0.01 to 1. The *min_weight_fraction_leaf* parameter represents the minimum weight fraction of samples in a leaf node, and the search range is from 0.01 to 0.2. The *ccp_alpha* parameter is a parameter used for cost complexity pruning. It balances the complexity of the DT and the fitting error on the training data, and the search range is from 0.01 to 0.2. After 500 iterations, the fitness of the model tends to be stable. The optimal values of different parameters are shown in Table 6.

**TABLE 6:** Comparison of key parameter values of DT.

Parameters	Intelligent optimization algorithms				
	BES	SSA	GWO	WOA	HHO
max_depth	6	16	20	8	3
min_samples_leaf	0.0747	0.0552	0.0594	0.1106	0.0100
max_features	0.0280	0.0469	0.3384	0.0105	0.0100
min_weight_fraction_leaf	0.0604	0.0617	0.2000	0.0105	0.0100
ccp_alpha	0.0736	0.0747	0.2000	0.0105	0.0100

**FIGURE 3:**
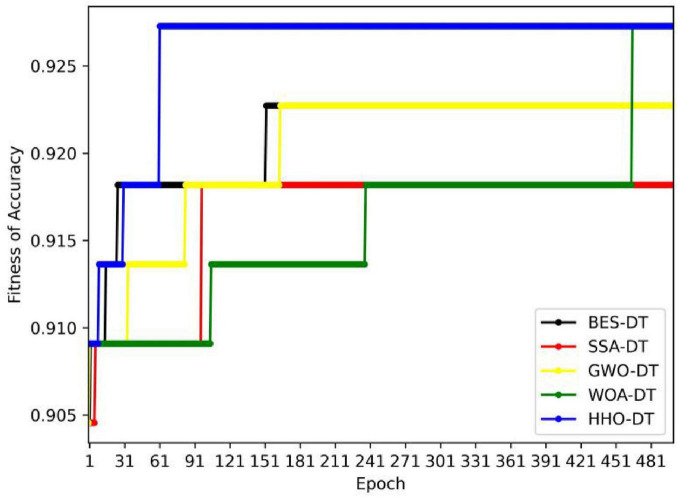
DT optimized by different intelligent optimization algorithms.

Figure 3 shows the changes in the accuracy of DT models based on different optimization algorithms (BES-DT, SSA-DT, GWO-DT, WOA-DT, HHO-DT) during the training process with the number of iterations when the number of iterative steps is 500. Overall, the accuracy of each model shows different trends during the iteration process. Some models quickly reach a high accuracy in the early stage and remain stable, while some models experience a more tortuous growth process before tending to be stable.

The accuracy of the HHO-DT model is relatively low in the early stage of iteration, about 0.905. However, with the progress of iteration, its accuracy gradually increases. It reaches about 0.918 at around the 30th iteration and remains stable at 0.927 subsequently. This indicates that the HHO has certain potential when optimizing the hyper parameters of the DT. Although its performance is not good in the early stage, it can effectively improve the performance of the model through continuous iteration and finally enable the model to achieve a high accuracy. The final accuracies of the GWO-DT and BES-DT models are 0.923; the final accuracies of the SSA-DT and WOA-DT models are 0.918. We further performed 10 rounds of verification on the HHO-DT, BES-DT, GWO-DT, SSA-DT, and WOA-DT algorithms, and calculated their average values. As shown in Supplementary Table 1, the HHO-DT algorithm achieves the highest accuracy.

In this study, we compared with grid search (GS) strategy and tuned five core parameters. The parameter ranges were consistent with the previous description, *max_depth* was set with a step size of 2, *min_samples_leaf* and *min_weight_fraction_leaf* with a step size of 0.01, *max_features* with a step size of 0.2, and *ccp_alpha* with a step size of 0.05.

**FIGURE 4:**
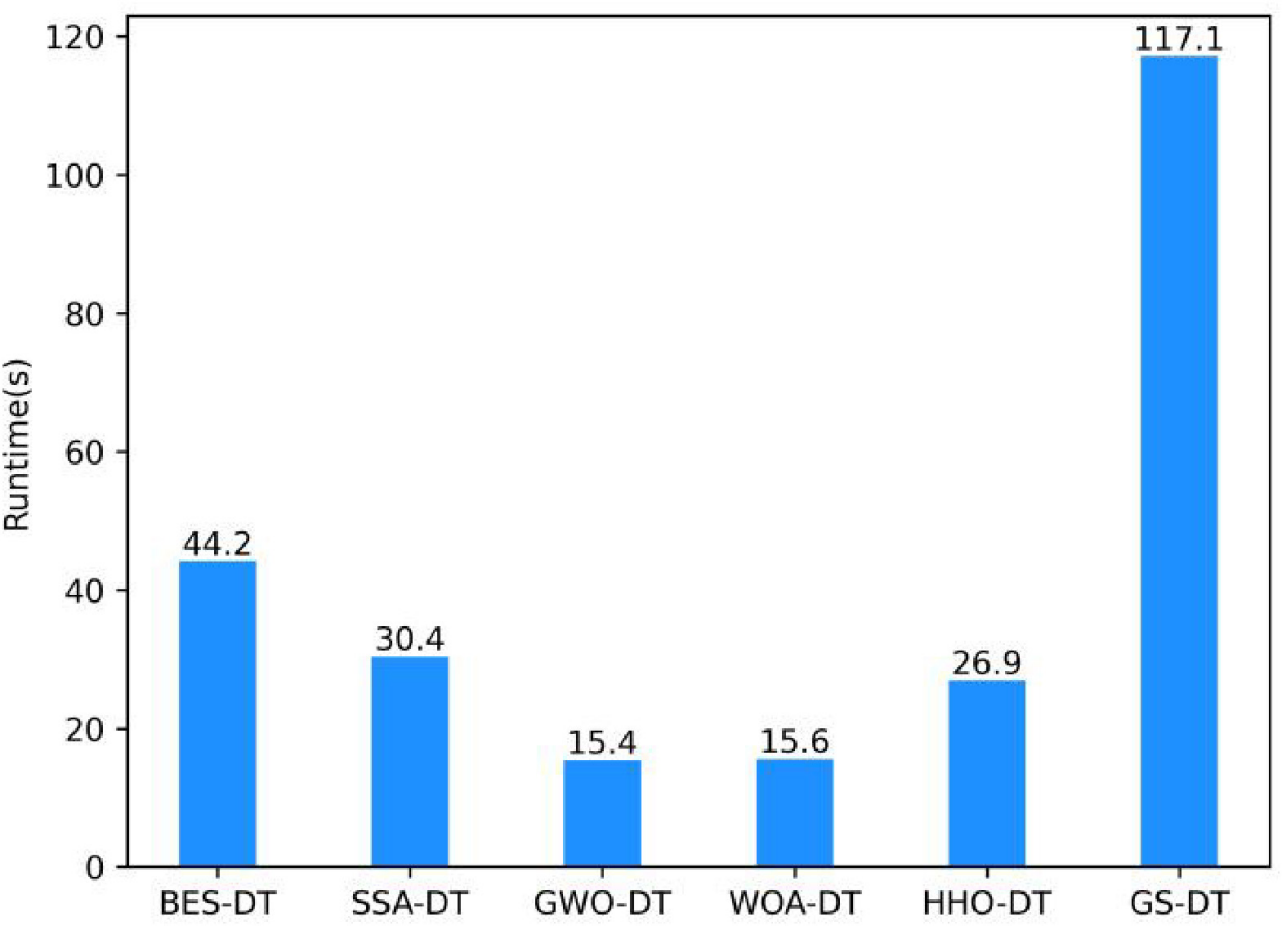
Comparison of running time of different optimization algorithms.

From Figure 4, it can be seen that the GWO-DT and WOA-DT have the shortest running times, which are 15.4 s and 15.6 s, respectively. These algorithms are suitable for scenarios requiring rapid parameter tuning. The running time of the HHO-DT algorithm is 26.9 s, which lies between that of the group search algorithm (BES at 44.2 s) and the individual search algorithm (SSA-DT at 30.4 s). The GS-DT takes 117.2 s, and as the step size becomes smaller, the grid search time will increase further. HHO-DT control the running time within a reasonable range while ensuring optimization effectiveness, making it particularly suitable for small-dataset scenarios. In contrast, although grid search can exhaustively enumerate parameter combinations, its computational cost is excessively high.

**FIGURE 5:**
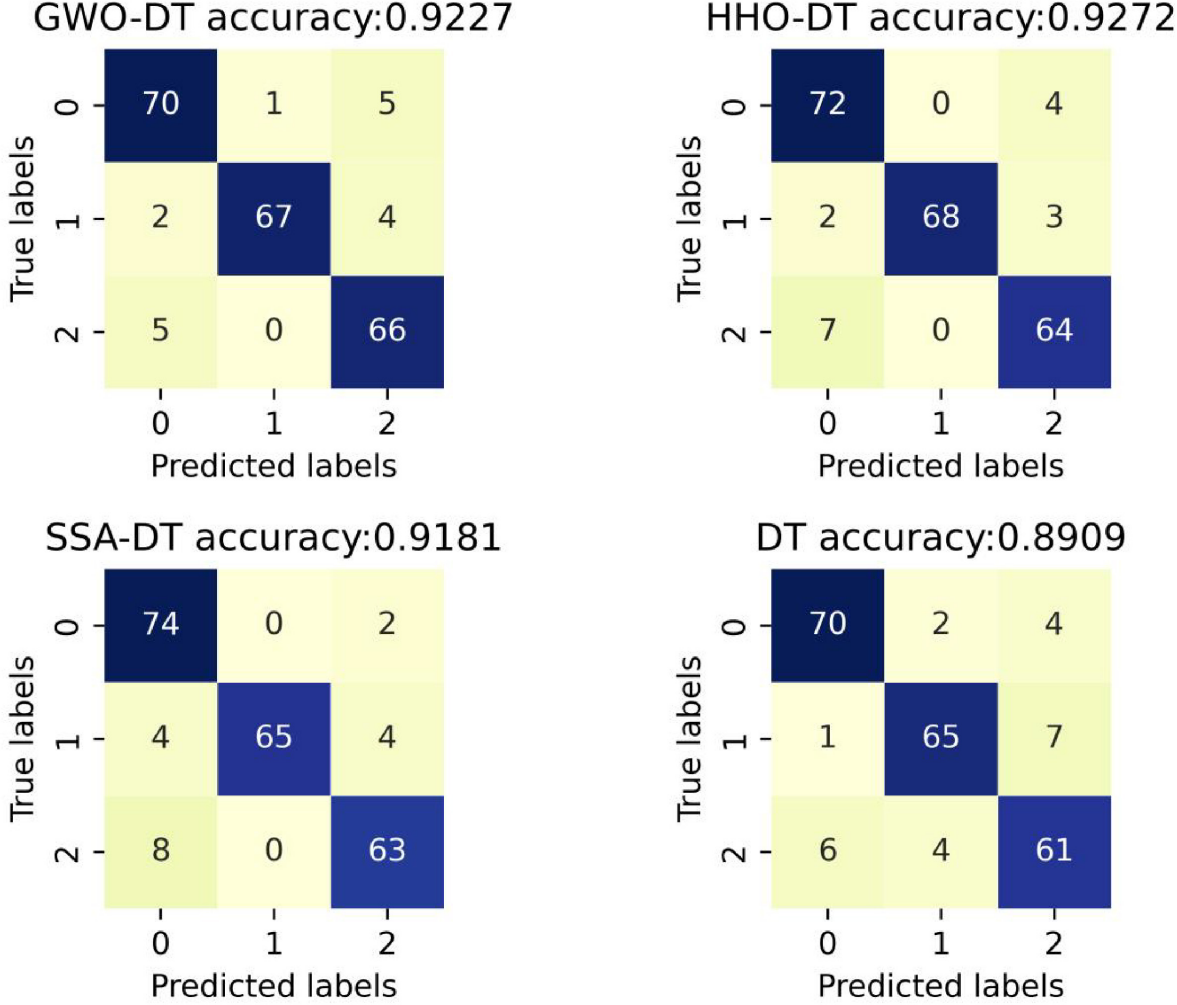
Confusion matrices in student stress classification.

**FIGURE 6:**
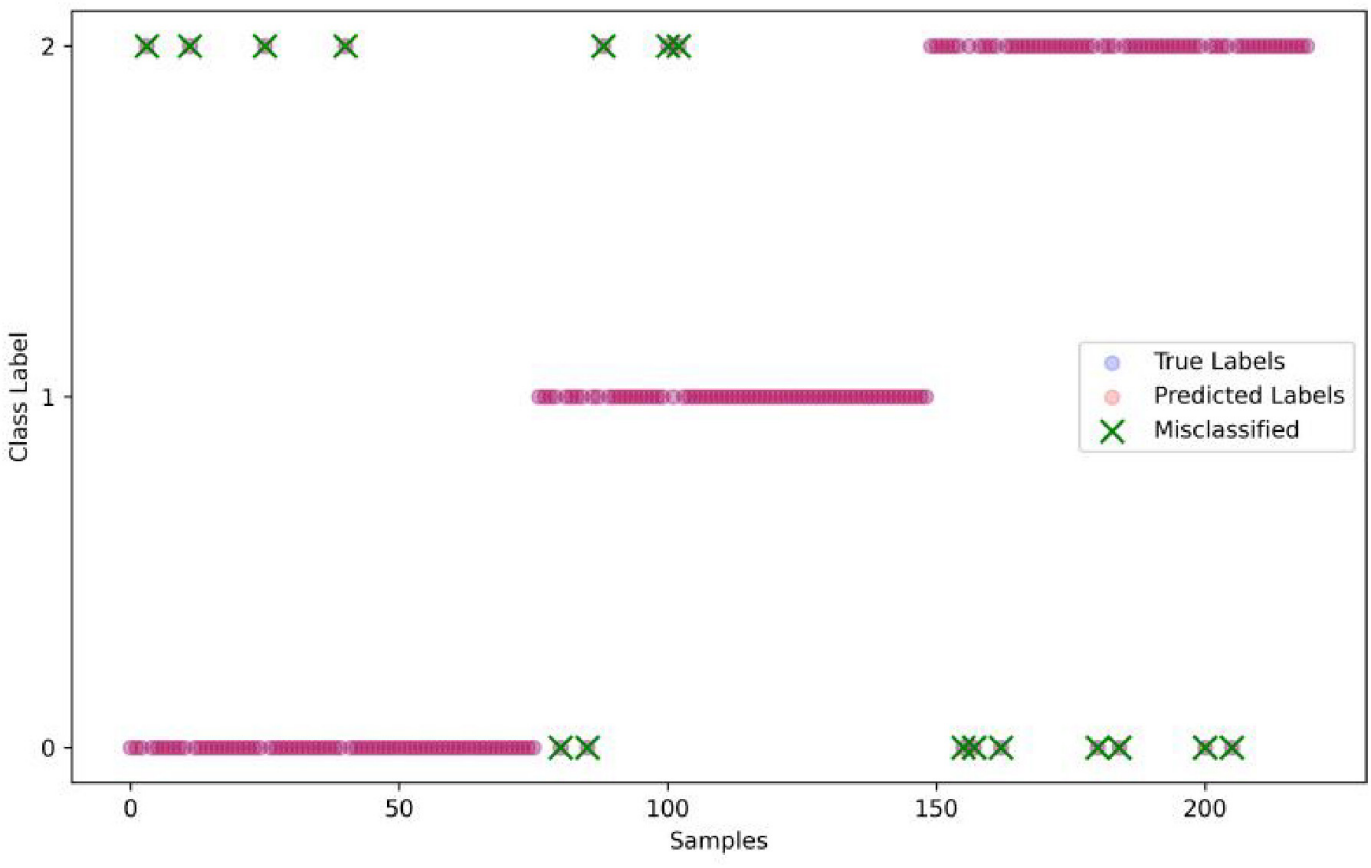
True and predicted labels with misclassified samples.

Based on the fitness iteration curves in Figure 3, the GWO-DT, HHO-DT, SSA-DT, and DT are selected for a comparative analysis using confusion matrices, and the results are shown in Figure 5. The accuracy of GWO-DT is 0.9227, that of HHO-DT is 0.9272, SSA-DT is 0.9181, and DT is 0.8909. It can be seen that the accuracies of the DT models improved by optimization algorithms are all higher than that of the original model, among which the HHO-DT has the highest accuracy. As is shown in Figure 6, in the HHO-DT model, only 16 samples are misclassified, which is the smallest number among the compared models, indicating that the HHO algorithm is quite effective in enhancing the classification performance of the DT model.

### 4.3 SHAP analysis of stress level

**FIGURE 7:**
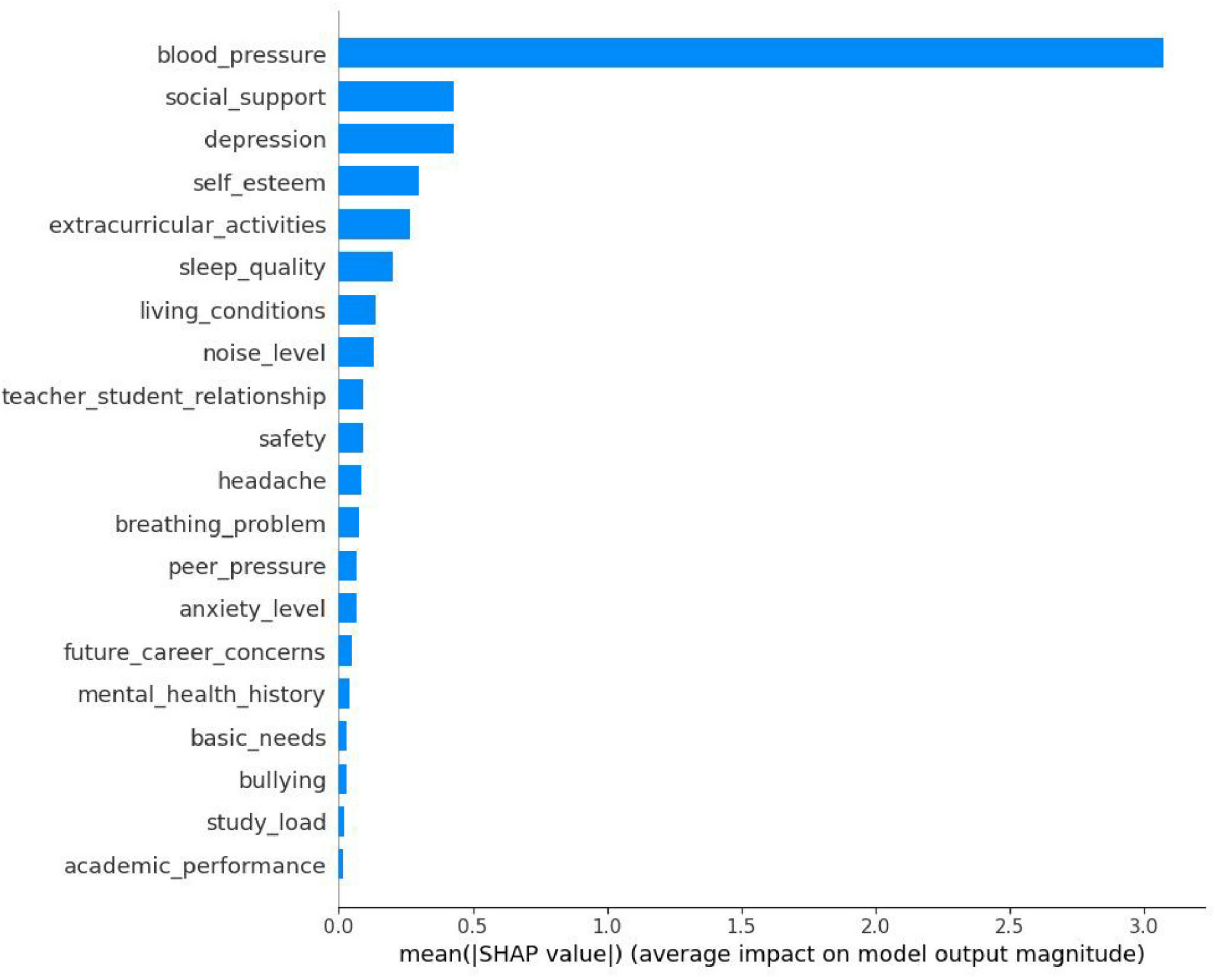
SHAP values of various features for predicting students’ stress levels.

As is shown in Figure 7, a bar chart of feature importance based on SHAP values, presenting the average impact degree of different features on the prediction results of students’ stress levels. It can be seen from the figure that the average SHAP value of *blood_pressure* is the highest, indicating that it has the greatest impact on the model’s prediction of students’ stress levels and is a key factor affecting students’ stress levels. Features such as *social_support*, *depression*, and *self_esteem* also have relatively high average SHAP values, indicating that they also play a relatively important role in the prediction of students’ stress levels. However, features such as study_load and academic_performance have relatively low average SHAP values and have a relatively small impact on the model’s prediction results.

**FIGURE 8:**
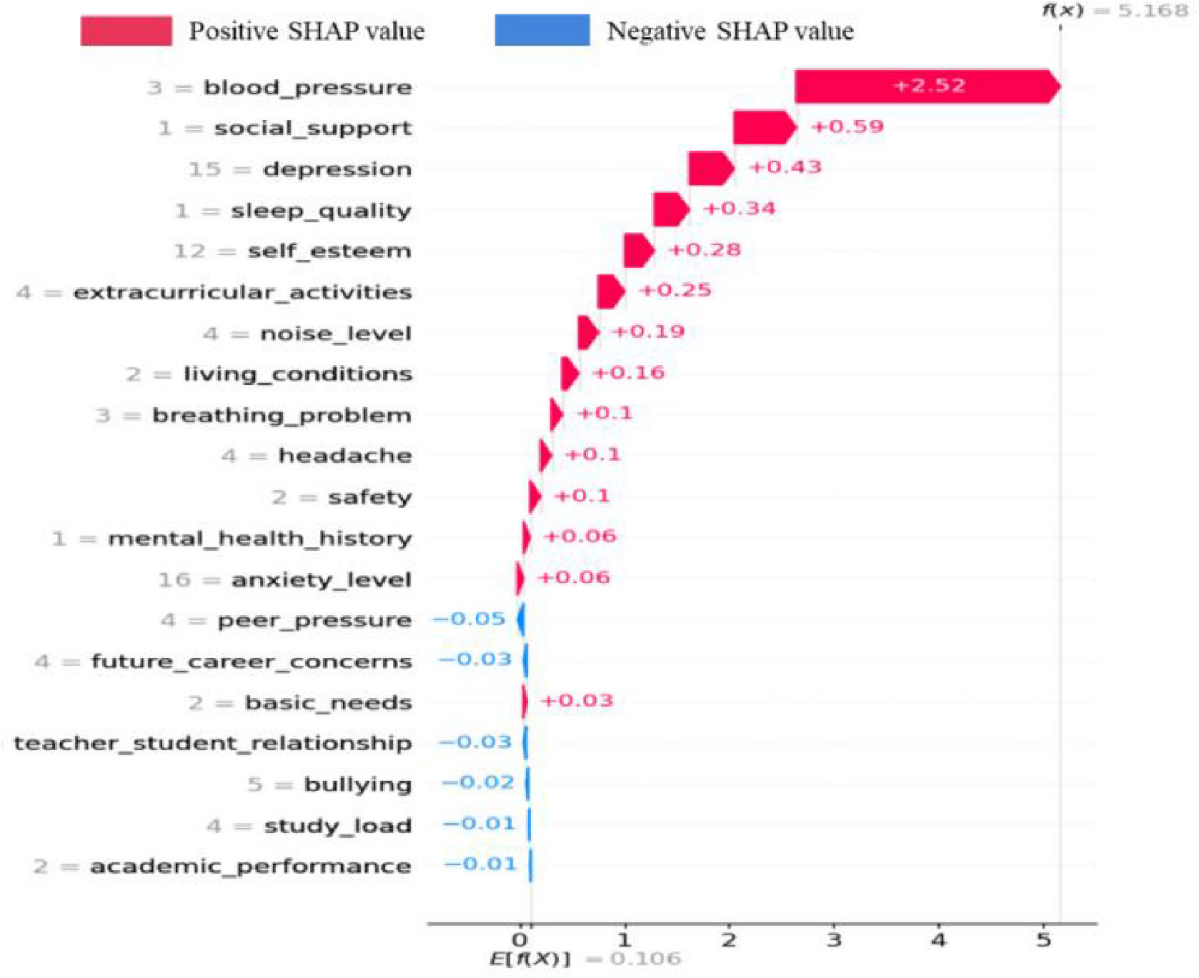
SHAP values of various features in a specific case.

As shown in Figure 8, it displays the SHAP values of various features for predicting students’ stress levels in a specific case. The f(x) = 5.168 on the far-right side of the figure is the final predicted value of the model after comprehensively considering all features. The values and arrows next to each feature indicate the impact of that feature on the prediction result. A positive value such as +0.52 for blood_pressure means that the feature increases the predicted value, that is, it drives the model to predict a higher stress level; a negative value such as −0.02 for bullying means that the feature decreases the predicted value, that is, it inhibits the model from predicting a higher students’ stress level. The larger the absolute value of the number, the greater the impact of the feature on the prediction result. Through this figure, we can intuitively understand the comprehensive impact of each feature on the prediction of students’ stress levels, which is helpful for analyzing which factors play a major role in the prediction of students’ stress, thus providing a reference basis for subsequent research and intervention measures on students’ stress.

**FIGURE 9:**
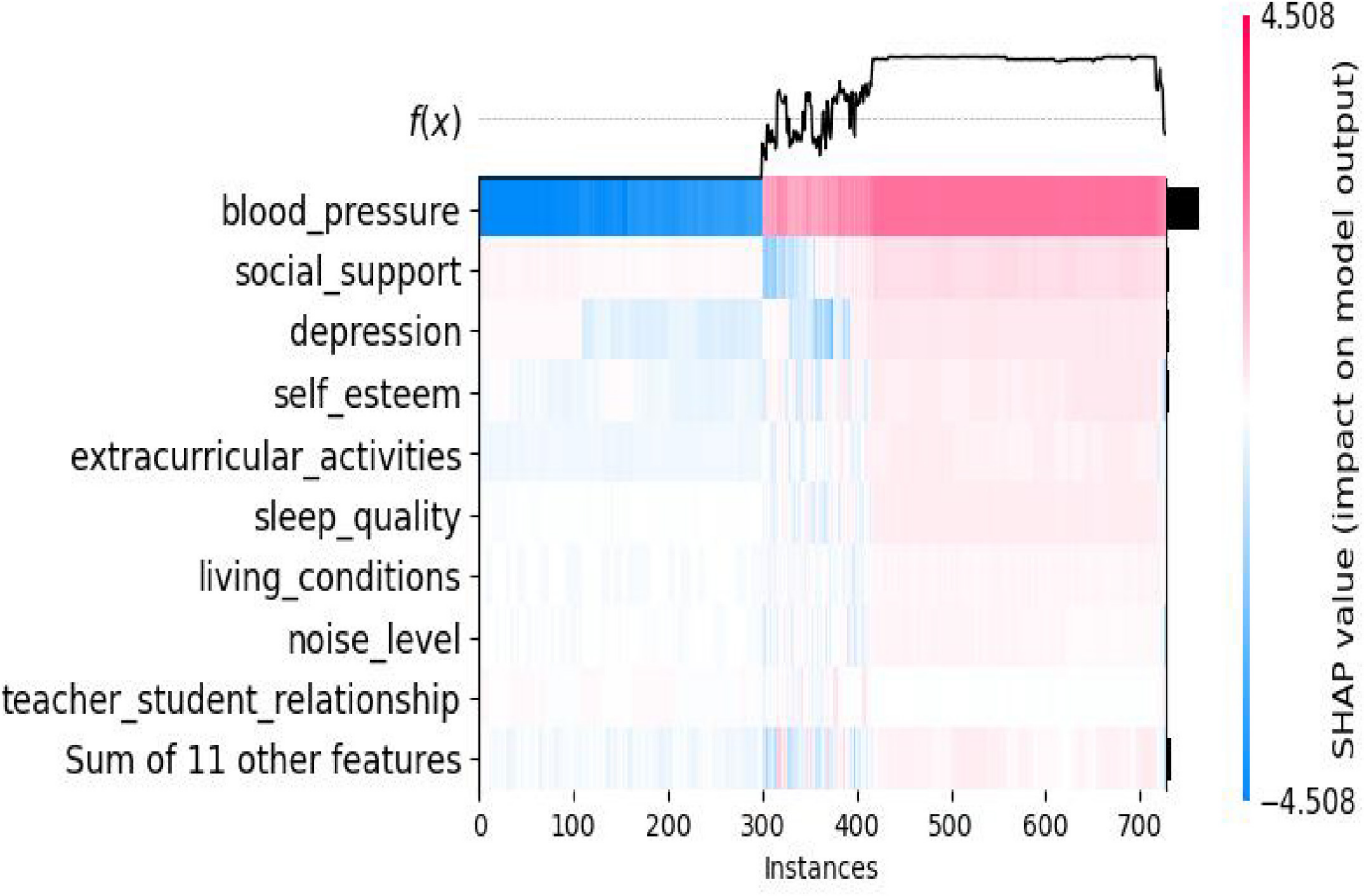
SHAP values of various features for multiple cases.

As shown in Figure 9, for some features such as *blood_pressure* and *social_support*, they show relatively dark colors in a large number of instances, indicating that these features have a relatively significant and widespread impact on the prediction of students’ stress levels in different samples. For the “Sum of 11 other features,” its color distribution is relatively light and scattered, suggesting that the sum of these 11 features has a relatively small impact on the model’s prediction.

To explore the role of social support in the mechanism underlying students’ stress, this study categorized participants into the high social support group (score ≥ 3) and low social support group (score ≤ 2) based on their social_support scores. Independent samples *t*-test was employed to compare feature differences between the two groups, with results presented in Table 7. At the conventional significance threshold of 0.05, statistically significant differences were observed in 12 features between the two groups. For instance, the high social support group exhibited significantly better *living_conditions* (*t* = 10.70, *p* < 0.001) and higher satisfaction with *basic_needs* (*t* = 5.77, *p* < 0.001) than the low social support group, while their noise exposure was significantly lower (*t* = 5.82, *p* < 0.001). These findings suggest a mutually reinforcing relationship between a favorable living environment and higher social support. However, no statistically significant differences were found in 7 features, including *teacher_student_relationship*, *depression*, and *study_load* (*p* > 0.05), highlighting the need for further investigation into the relationships between these factors and social support in future research.

**TABLE 7:** *T*-test for feature differences between high and low social support groups.

Field name	*t*-value	*p*-value	Significance
living_conditions	10.70	1.09E–24	Yes
noise_level	5.82	9.34E–09	Yes
basic_needs	5.77	1.08E–08	Yes
mental_health_history	5.24	2.14E–07	Yes
breathing_problem	5.23	2.26E–07	Yes
sleep_quality	−4.54	6.62E–06	Yes
future_career_concerns	4.55	6.63E–06	Yes
Bullying	4.44	1.01E–05	Yes
anxiety_level	3.75	1.94E–04	Yes
self_esteem	−3.66	2.74E–04	Yes
extracurricular_activities	−3.01	2.74E–03	Yes
Safety	1.97	4.92E–02	Yes
teacher_student_relationship	1.90	5.83E–02	No
Depression	1.83	6.79E–02	No
study_load	1.48	1.38E–01	No
academic_performance	−1.17	2.40E–01	No
peer_pressure	0.70	4.84E–01	No
blood_pressure	0.66	5.11E–01	No
Headache	0.00	9.98E–01	No

## 5 Discussion and Conclusion

### 5.1 Deep exploration of feature impacts

Features with high SHAP values, as shown in Figures 6–8, such as *social_support* and *depression*, provide important directions for formulating student stress intervention strategies. Strengthening students’ social support networks can be achieved through various means. For example, organizing social activities to promote communication and mutual assistance among students; establishing a good communication mechanism between teachers and students so that students can receive timely support and help when encountering problems. To pay attention to students’ mental health status, mental health education courses can be carried out to improve students’ psychological adjustment ability, and psychological counseling services can be provided to detect and solve students’ psychological problems in a timely manner. By improving these factors, it is expected to effectively reduce students’ stress levels. Arya et al. (2024) identified through correlation analysis that *self_esteem* and *sleep_quality* exert significant negative impact on students’ stress, while *bullying* has a significant positive impact on students’ stress. In contrast, the present study, based on quantitative analysis using the SHAP model, demonstrates that physiological factors are the most influential predictors of stress, followed by *social_support* and *depression*.

Elevated *blood pressure* aligns with the physiological outcomes of Lazarus’ transactional model (Obbarius et al., 2021). After primary appraisal (evaluating a stressor as threatening), secondary appraisal (assessing coping resources) triggers physiological responses like increased *blood pressure*, validating the theory’s focus on mind-body interactions in stress. The SHAP emphasis on *social_support* reflects Lazarus’ secondary appraisal process. Low social support signals insufficient coping resources, a key factor in stress evaluation. Individuals appraising their environment as lacking supportive relationships (e.g., friends, family) are more likely to perceive stressors as unmanageable.

In SHAP analysis, *study_load* and *academic_performance* have the least impact on student stress in Nepal. This outcome is shaped by the interplay of educational systems, social culture, and economic conditions. Compared to country like China, Nepal’s low academic stress stems from a balance between limited educational resources and societal tolerance. Local communities prioritize basic education completion over individual academic excellence, with parents focusing more on children’s wellbeing than exam results. Additionally, due to limited access to higher education, only a small fraction of students pursuing university education face intense competition.

Based on the results of SHAP analysis, there are likely interactions between features. For example, good social support may reduce the impact of depression on students’ stress levels, while poor social support may exacerbate the negative impact of depression. Although the current research has revealed the individual impacts of each feature, the interactions between features have not been deeply explored. In the future, more complex data analysis methods, such as constructing interaction effect models, can be used to deeply study these interactions and further improve the understanding of the student stress prediction mechanism. By delving deeper into the complex relationships between features, more targeted and precise intervention measures can be developed to improve the intervention effect.

We analyzed the misclassified cases. There is a case where the corresponding stress level is stress-2, with both depression and social support rated as 1. The model misclassified it as stress-1. Judging from a single feature, although there is a lack of social support, considering its depression feature and other dimensional combinations, the model classified it as moderate stress. In the actual data environment, the interaction between them and numerous other features may have changed the final prediction result. In the future, we can try to construct new composite features or transform the existing ones to better capture the potential information in the data. For example, combining relevant physiological, psychological and social features to form more representative comprehensive features that can help the model judge students’ stress levels more accurately.

### 5.2 Measures to alleviate students’ stress

It is essential to establish a robust support system, wherein schools and families collaborate to create a strong network that provides necessary psychological counseling and support services (Ruiz and Lopez, 2024). Students should be guided to perceive stress accurately, encouraging them to analyze situations calmly and view stressful circumstances as opportunities to develop their abilities, rather than succumbing to fear or avoidance. Universities can use our prediction model’s results to identify students who at high risk of stress, and can allocate mental health resources more effectively, focusing on students who need it most.

Through targeted training and education, students can be equipped with effective coping strategies (English and Chi, 2020). Instruction in emotional regulation skills, such as deep breathing, relaxation techniques, and meditation, can enable students to swiftly regain composure during emotional turmoil, thereby preventing them from being overwhelmed by negative emotions and allowing them to manage pressure more rationally. Furthermore, through education on resilience, students can cultivate a tenacious character by facing repeated challenges. They should be taught that failure serves as a stepping stone to success, and that each setback is an opportunity to rise again and grow stronger, ultimately enhancing their psychological endurance.

To enhance educational outcomes, it is essential to improve teaching methods and evaluation systems, reduce unnecessary academic burdens, and foster a more relaxed learning atmosphere (Mahalingam et al., 2018; Tom, 2022). This can be achieved through the development of personalized learning plans that align with students’ individual learning rhythms, strengths, and areas for improvement. It is important to allocate study time effectively, emphasizing key concepts and challenging topics while avoiding rote practice of questions. This approach aims to enhance learning efficiency and alleviate academic pressure. Students should be encouraged to explore learning strategies that resonate with their preferences; for instance, visual learners might benefit from mind maps to bolster memory retention, while auditory learners could utilize audiobooks to facilitate their studies. Such tailored methods can stimulate interest in learning and improve academic performance. Furthermore, it is crucial for students to actively communicate any learning challenges to their teachers and provide timely feedback on their progress. This collaboration allows educators to adjust their teaching strategies effectively, fostering a mutually beneficial learning environment.

Regular mental health lectures and activities should be organized to bolster students’ psychological resilience (Arif et al., 2021). Additionally, a variety of team activities, such as group projects and club competitions, can promote communication and collaboration among students, helping them forge meaningful friendships, enhance social skills, and alleviate social anxiety through interaction. For students facing significant social challenges, timely intervention by professional psychological counselors can be invaluable. Through one-on-one tutoring, these counselors can help students navigate their emotions, rebuild confidence, and develop healthy interpersonal relationships. Finally, supporting students in exploring personal interests and hobbies—such as painting, music, and reading—can provide them with an immersive escape from their worries, enriching their spiritual lives and adding vibrancy to their experiences.

### 5.3 Comparison with deep learning-based approaches

The HHO-DT model offers a clear classification logic, yet its universality and accuracy need further verification with more data. Among various machine learning algorithms, the DT algorithm shows good performance in accuracy. Meanwhile, artificial intelligence has made significant advancements in stress prediction, with traditional machine learning and deep learning methods each demonstrating distinct characteristics. Compared to machine learning, deep learning approaches such as CNN (Tian, 2022), LSTM (Hafeez and Shakil, 2023) show greater potential in processing complex multimodal data (e.g., physiological signals, text, behavioral logs) (Morgan, 2017). However, they rely on large-scale datasets and high computational resources while suffering from insufficient model transparency. As shown in Arya et al. (2024), in terms of algorithm performance, the Naive Bayes model performed the best, achieving a test accuracy of 0.90, while the SVM model performed the worst. In contrast, the HHO-DT model proposed in our study has a test accuracy improved to 0.927, representing an increase of 2.7% points. This demonstrates a significant advantage in prediction accuracy.

Additionally, deep learning excels in automated feature extraction, capable of capturing latent patterns from raw data (e.g., heart rate variability, social media text), but faces challenges like ethical risks and high deployment costs. The core differences between this study and deep learning lie in data scale, interpretability, and applicable scenarios. The lightweight HHO-DT model is suitable for resource-constrained environments with transparent decision logic, facilitating educators’ intervention strategy formulation. In contrast, deep learning can uncover more complex nonlinear relationships with sufficient data but requires balancing performance and interpretability. Future research could explore hybrid models, for example, using CNN to extract physiological signal features before combining with optimized DT for classification, to integrate automation and interpretability. Ultimately, method selection should consider data characteristics, interpretability requirements, and resource constraints. This study provides an efficient and reliable solution for medium-scale scenarios, while deep learning still holds vast development potential in multimodal big data applications.

### 5.4 Future research directions

To improve the performance of models in student stress prediction, we can attempt to introduce new machine learning algorithms, such as neural network models in deep learning. These models have stronger non-linear fitting capabilities and can better capture the complex relationships in the data. We can also improve existing algorithms. For example, adjust the growth strategy of DT, optimize the combination method of ensemble learning models, etc., to enhance the accuracy and stability of the models. Regarding the problem of sample imbalance, data sampling techniques such as oversampling, undersampling, or synthetic minority over-sampling technique (SMOTE) can be adopted to balance the sample sizes of different stress categories and improve the model’s ability to identify minority classes.

Future research can further expand the exploration of features affecting students’ stress levels. More psychological factors, such as students’ personality traits and coping styles, can be considered, as well as environmental factors like family environment and school atmosphere. A variety of data analysis methods, such as structural equation modeling and Bayesian networks, can be used to deeply study the interactions between features. These methods can more accurately reveal the causal relationships and complex network structures among variables, providing stronger theoretical support for the formulation of intervention measures. At the same time, longitudinal research methods can be combined to track the changes in students’ stress levels, dynamically analyze the relationships between features, and improve the timeliness and accuracy of student stress prediction. And, transformer can also be applied to analyze long sequence information such as students’ behavioral data and changes in psychological states. We can explore its potential in capturing complex features and long term trends, and compare it with traditional machine learning models to evaluate its advantages and applicability. By integrating real-time monitoring technologies and leveraging Internet of Things (IoT) devices such as wearables, we can achieve dynamic monitoring and early warning of students’ stress levels. Additionally, we can develop or integrate existing mobile health applications to enable students to conveniently record information about their psychological feelings and daily activities. Through interaction with the prediction model, students can achieve self-monitoring and management of their stress.

In the future, we will actively collaborate with multiple schools and educational institutions to collect large scale student data covering students from different regions, age groups, and disciplines. By conducting experiments on data from different institutions, we can evaluate the model’s performance in different environments, identify the problems and limitations of the model, and then make improvements and optimizations.

## 6 Limitations of the data

The dataset sourced from Kaggle presents several limitations. First, the sample lacks representativeness and exhibits geographic and cultural bias. The data is predominantly collected from Nepal and does not include regions such as the United States or other countries. Second, the dataset only covers individuals aged 15–24, making it difficult to reflect characteristics of younger groups like elementary school students. Although we conducted subgroup analysis focusing on the social support dimension to explore potential differences in stress levels among groups with varying access to social support, the study still faces a key constraint: due to the lack of an explicit age variable in the dataset, we cannot perform more granular stratification by academic stages. This limitation prevents the full manifestation of differences in how social support moderates stress across different developmental stages. Additionally, critical variables are missing, including key social determinants of student health such as household income, parental education levels, and community resources, which leads the model to overlook the impacts of structural inequalities. Finally, the dataset lacks temporal dynamics, as it is cross-sectional and fails to capture longitudinal changes in student conditions over time.

Although the proposed model achieves an accuracy of 0.928 on the dataset used in this study, its generalization ability still requires further discussion, primarily due to limitations imposed by differences across academic stages as well as regional and cultural disparities. Meanwhile, student stress prediction involves sensitive information such as psychological states and physiological indicators, and model decisions may affect the allocation of intervention resources, thus, attention must also be paid to privacy protection and bias mitigation.

## Data Availability

The original contributions presented in the study are included in the article/Supplementary material, further inquiries can be directed to the corresponding author.
