# Supplementary material for: Students’ stress prediction and explainable analysis based on improved decision trees
Source: Front Psychol. 2026 Jan 2;16:1684529. doi: 10.3389/fpsyg.2025.1684529 (PMC12808363; doi:10.3389/fpsyg.2025.1684529)
Supplement: Supplementary file 1 [file Table_1.DOCX]

We also conducted 10 rounds of verification on HHO-DT, BES-DT, GWO-DT, SSA-DT, and WOA-DT, and took the average values. The HHO-DT algorithm has the highest accuracy rate.

Supplementary Table 1. Comparison of the Average Accuracy Rates of Various Models in 10 Rounds of Verification

| Rounds | Models | | | | |
| --- | --- | --- | --- | --- | --- |
|  | BES-DT | SSA-DT | GWO-DT | WOA-DT | HHO-DT |
| Round-1 | 0.932 | 0.914 | 0.909 | 0.927 | 0.927 |
| Round-2 | 0.918 | 0.932 | 0.918 | 0.918 | 0.927 |
| Round-3 | 0.927 | 0.909 | 0.923 | 0.918 | 0.927 |
| Round-4 | 0.927 | 0.918 | 0.923 | 0.923 | 0.918 |
| Round-5 | 0.923 | 0.914 | 0.923 | 0.927 | 0.927 |
| Round-6 | 0.923 | 0.923 | 0.923 | 0.932 | 0.932 |
| Round-7 | 0.932 | 0.918 | 0.923 | 0.923 | 0.932 |
| Round-8 | 0.923 | 0.923 | 0.918 | 0.927 | 0.927 |
| Round-9 | 0.923 | 0.927 | 0.918 | 0.923 | 0.927 |
| Round-10 | 0.932 | 0.918 | 0.923 | 0.923 | 0.932 |
| Average | 0.926 | 0.920 | 0.920 | 0.924 | **0.928** |
